# Reactogenicity and immunogenicity of the second COVID-19 vaccination in patients with inborn errors of immunity or mannan-binding lectin deficiency

**DOI:** 10.3389/fimmu.2022.974987

**Published:** 2022-09-14

**Authors:** Lisa Göschl, Daniel Mrak, Katharina Grabmeier-Pfistershammer, Karin Stiasny, Helmuth Haslacher, Lisa Schneider, Thomas Deimel, Felix Kartnig, Selma Tobudic, Daniel Aletaha, Heinz Burgmann, Michael Bonelli, Winfried F. Pickl, Elisabeth Förster-Waldl, Clemens Scheinecker, Matthias Gerhard Vossen

**Affiliations:** ^1^ Division of Rheumatology, University Clinics of Internal Medicine III, Medical University of Vienna, Vienna, Austria; ^2^ Institute of Immunology, Centre for Pathophysiology, Infectiology and Immunology, Medical University of Vienna, Vienna, Austria; ^3^ Center for Virology, Medical University of Vienna, Vienna, Austria; ^4^ Department of Laboratory Medicine, Medical University of Vienna, Vienna, Austria; ^5^ Division of Infectious Diseases and Tropical Medicine, Department of Medicine I, Medical University of Vienna, Vienna, Austria; ^6^ Division of Neonatology, Pediatric Intensive Care and Neuropediatrics with Centre for Congenital Immunodeficiencies & Jeffrey Modell Center Vienna, Department of Pediatrics and Adolescent Medicine, Medical University of Vienna, Vienna, Austria

**Keywords:** autoimmune diseases, B-lymphocytes, COVID-19, vaccination, autoimmune diseases, T-lymphocites

## Abstract

**Background:**

Patients with inborn errors of immunity (IEI) are at increased risk for severe courses of SARS-CoV-2 infection. COVID-19 vaccination provides effective protection in healthy individuals. However, it remains unclear whether vaccination is efficient and safe in patients with constitutional dysfunctions of the immune system. Thus, we analyzed the humoral response, adverse reactions and assessed the disease activity of the underlying disease after COVID-19 vaccination in a cohort of patients suffering from IEIs or mannan-binding lectin deficiency (MBLdef).

**Methods:**

Vaccination response was assessed after basic immunization using the Elecsys anti-SARS-CoV-2 S immunoassay and *via* Vero E6 cell based assay to detect neutralization capabilities. Phenotyping of lymphocytes was performed by flow cytometry. Patient charts were reviewed for disease activity, autoimmune phenomena as well as immunization status and reactogenicity of the vaccination. Activity of the underlying disease was assessed using a patient global numeric rating scale (NRS).

**Results:**

Our cohort included 11 individuals with common variable immunodeficiency (CVID), one patient with warts hypogammaglobulinemia immunodeficiency myelokathexis (WHIM) syndrome, two patients with X-linked agammaglobulinemia (XLA), one patient with Muckle Wells syndrome, two patients with cryopyrin-associated periodic syndrome, one patient with Interferon-gamma (IFN-gamma) receptor defect, one patient with selective deficiency in pneumococcal antibody response combined with a low MBL level and seven patients with severe MBL deficiency. COVID-19 vaccination was generally well tolerated with little to no triggering of autoimmune phenomena. 20 out of 26 patients developed an adequate humoral vaccine response. 9 out of 11 patients developed a T cell response comparable to healthy control subjects. Tested immunoglobulin replacement therapy (IgRT) preparations contained Anti-SARS-CoV-2 S antibodies implicating additional protection through IgRT.

**Summary:**

In summary the data support the efficacy and safety of a COVID-19 vaccination in patients with IEIs/MBLdef. We recommend evaluation of the humoral immune response and testing for virus neutralization after vaccination in this cohort.

## Highlights

### What is already known about this subject?

- There is an ever growing body of literature describing COVID-19 vaccine response in IEI. However there is a relative lack of studies of patients with specific pathway defects. Reports are mostly about patients suffering from primary and secondary hypogammaglobulinemia.
- We found no reports about worsening of pre-existing autoimmune disease in patients with IEI

### What does this study add?

- This study further supports the good humoral and cellular immunogenicity of the available vaccines also in patients with IEI. To our knowledge, we are the first group to report no increased autoimmune phenomena or worsening of disease activity after vaccination in patients suffering from IEI or MBL deficiency.

### How might this impact on clinical practice?

- COVID-19 vaccination is safe and efficient in patients with IEI or MBL deficiency. Therefore, COVID-19 vaccination is highly recommended in patients with IEI or MBL deficiency.
- IEIs show a high pathogenetic diversity, therefore we strongly recommend testing of the humoral immune response in combination with testing for virus neutralization in all patients.

## Introduction

Currently the most important measure to reduce disease burden and combat the SARS-CoV-2 pandemic is the vaccination of the general population and particularly of individuals at increased risk for a severe course of COVID-19 disease. Patients suffering from inborn errors of immunity (IEIs) and especially those with reduced or dysregulated humoral immunity such as common variable immunodeficiency (CVID), may be at increased risk for severe and critical COVID-19 infection, although oligosymptomatic SARS-CoV-2 infections have been reported as well (1–5). Protection through vaccination has been shown to be effective and safe in the general population. In patients suffering from secondary immunosuppression an additional booster vaccination showed an increase in humoral and cellular response (6). There is an ever increasing amount of data available regarding the safety and efficiency of COVID-19 vaccines in IEI/MBLdef patients. Vaccination with BNT162b2 (Corminaty^®^, Pfizer-BioNTech) as well as mRNA-1273 (Spikevax^®^, Moderna) and Ad26.COV2.S (COVID-19 Vaccine Janssen^®^, Johnson&Johnson) has been shown to produce humoral and/or cellular responses in several collectives of patients suffering from inborn errors of immunity irrespective of the underlying pathology (7–19). We intend to contribute to this body of knowledge and provide additional insight regarding vaccine reactogenicity.

The objective of this study was to evaluate the humoral and cellular immunogenicity and patient reported adverse events as well as the impact of vaccination on the underlying disease activity of different SARS-CoV-2 vaccines in immunocompromised patients as well as in a group of individuals showing decreased mannan-binding lectin levels.

## Patients and methods

26 patients from the interdisciplinary outpatient clinic for patients with IEIs at the Division of Infectious Diseases and Tropical Medicine and the Division of Rheumatology at the Medical University of Vienna were enrolled in this study. Healthy adults, twice vaccinated with BNT162b2, served as sex and age matched healthy control (HC) group. All patients gave their written informed consent to retrospective data analysis as well as biobanking of blood samples. Serum samples were collected during routine check-up visits after the second vaccination and consecutively stored at the biobank of the Medical University of Vienna a centralized facility for the preparation and storage of biomaterial with certified quality management (International Organization for Standardization (ISO) 9001:2015) (20). Blood samples were collected 105.5 (IQR: 75-129.5) days after the second vaccination. All patients received their second vaccination between March 2021 and August 2021. Ethical approval for this study was granted by the ethics committee of the Medical University of Vienna, Austria (1073/2021; 1075/2021; 1448/2019). Patients or the public were not involved in the design, or conduct, or reporting, or dissemination plans of our research.

### Humoral immune response upon SARS-CoV-2 vaccination

For anti-SARS-CoV-2 antibody testing the Elecsys Anti-SARS-CoV-2 S immunoassay was used for the quantitative determination of total antibodies to the receptor binding domain (RBD) of the viral spike (S) protein (21). The quantification range of this test is between 0.4 and 2500.0 U/mL. Anti-SARS-CoV-2 antibody levels reported by this test may be converted 1:1 to BAU/ml (22). For easy interpretation, we used BAU/ml throughout the manuscript. Anti-nucleocapsid antibodies produced in response to infection but not vaccination with any of the available mRNA- or vector-based vaccines were detected by the qualitative Elecsys anti-SARS-CoV-2 immunoassay. As recommended by the manufacturer, results >1,000 COI (cut-off index) were considered positive. Tests were performed on a Cobas e801 analyser (Roche Diagnostics, Rotkreuz, Switzerland) at the Department of Laboratory Medicine, Medical University of Vienna (certified acc. to ISO 9001:2015 and accredited acc. to ISO 15189:2012). Routinely assessed immunoglobulin levels were collected from patients’ charts. Neutralizing capability of the patients’ anti-spike protein antibodies was measured in a live virus neutralization assay at the Center for Virology of the Medical University of Vienna as described previously, using a SARS-CoV-2 virus strain isolated early during the pandemic (D614G mutation in the spike; GISAID accession number: EPI_ISL_438123) and Vero E6 cells. Neutralizing test (NT) titers were expressed as the reciprocal of the serum dilution required for protection against virus-induced cytopathic effects. NT titers ≥10 were considered positive (23). For the analysis of immunoglobulin preparations, the batches were diluted to 2 mg/mL IgG content and then analysed using Anti-SARS-CoV-2-QuantiVac-ELISA^®^ (EUROIMMUN, Lübeck, Germany) according to the manufacturer’s protocol.

### Quantification of peripheral lymphocytes and serum mannan-binding lectin (MBL) levels

Values of lymphocyte counts in peripheral blood as well as serum MBL levels were collected from patients’ charts. Analyses of whole blood were performed within 24h after blood drawing at the Institute of Immunology at the Medical University of Vienna. In short, samples were stained with indicated fluorescence labeled antibodies and lysed using NM-Lysis (Nordic MUbio) prior to flow cytometry analysis. Cells were gated by the expression of the pan-leukocyte marker CD45 (HI30) and granulocytes, monocytes and lymphocytes were separated by FSC/SSC and by using a combination of the following antibodies CD3 (UCHT1), CD4 (RPA-T4), CD8 (RPA-T8), CD19 (HIB19), CD20 (2H7), CD21 (555422), CD38 (303504), CD27 (302806), CD10 (312210), IgM (709-136-073) and IgD (H15501). Flow cytometry was performed using a Navios Flow Cytometer, Beckman Coulter^®^ and data were further analyzed using the Kaluza software, Beckman Coulter^®^. Characterization of class switched and non class switched B cells was achieved by gating for CD19^+^ B cells, followed by a CD27^+^ gating and finally differentiation *via* IgD^+^ and IgD^-^. Results were expressed as absolute cell count (cell/µl) among total lymphocytes. MBL serum levels (ng/ml) were assessed using the Hycult Biotech Human MBL ELISA Kit (Uden, The Netherlands).

### T cell response

For T cell stimulation, PepMix SARS-CoV-2 peptide pools for wild type (WT) were acquired from JPT (Berlin, Germany). The spike (S) peptides are split into sub-pools S1 (aa 1–643) and S2 (aa 633–1273) for wild type (WT). Peptides were dissolved in dimethyl sulfoxide and diluted in AIM-V medium for use in enzyme-linked immunosorbent spot (ELISpot) assays as described previously (6). For *ex vivo* T cell IFN-γ ELISpot assay, PBMCs from test subjects after the 2^nd^ vaccination were thawed and processed subsequently within one day. A total of 1–2×10^5^ cells per well were incubated with SARS-CoV-2 peptides (2 µg/mL; duplicates), AIM-V medium (negative control; 3–4 wells) or phytohemagglutinin (PHA) (L4144, Sigma; 0·5 µg/mL; positive control) in 96-well plates coated with 1.5 µg anti-IFN-γ (1-D1K, Mabtech) for 24 hours. After washing, spots were developed with 0.1 µg biotin-conjugated anti-IFN-γ (7-B6-1, Mabtech), streptavidin-coupled alkaline phosphatase (Mabtech, 1:1000) and 5-bromo-4-chloro-3-indolyl phosphate/nitro blue tetrazolium (Sigma). Spots were counted using a Bio-Sys Bioreader 5000 Pro-S/BR177 and Bioreader software generation 10. Data were processed as spot-forming cells (SFCs) per 10^6^ PBMCs after subtracting the spots from the negative control (mean spot numbers from three to four unstimulated wells).

### Assessment of the reactogenicity and Patient Global Assessment (PGA) of disease activity

Patients were regularly interviewed using a structured questionnaire regarding various autoimmune phenomena as well as immunization status and reactogenicity of the vaccination. Activity of the underlying disease was evaluated retrospectively using a patient global NRS (numeric rating scale) ranging from 0 to 10 with 0 denominating no disease activity and 10 the highest comprehendible disease activity. Furthermore, we examined the patients’ side effects after the first vaccination and after the second vaccination as well as anti-inflammatory drug usage.

### Statistical analysis

Continuous parameters are presented as mean with standard deviation or as median with interquartile range according to their distribution. Multivariable logistic regression was performed to identify variables associated with neutralizing activity. Variable selection was according to the expected relevance (IgG, peripheral B-cells, type of vaccine, MBL deficiency). Linear regression of week 4 and month 6 of anti-SARS-CoV-2 S antibody levels measured in the healthy control group after the second immunization was performed using Excel^®^ software (Microsoft^®^ Office 365). We thus generated interpolated anti-SARS-CoV-2 S antibody levels at the same time after vaccination as the measured anti-SARS-CoV-2 S antibody levels of the IEI/MBLdef patient cohort. “R” version 4.0.3 was used for the entire statistical analysis. P-values were calculated with an unpaired two tailed Student’s t-test or ANOVA. The following packages were utilized: “ggplot2” and “sjPlot” for creating plots and “tableone” to create the peripheral leucocyte analysis table. Lists, heat maps and plots were prepared using the Excel^®^ software (Microsoft^®^ Office 365) or GraphPad Prism 9.2.0.

## Results

### Patient characteristics

26 adults were included in this analysis, consisting of 19 patients with IEIs according to the recent IUIS classification (1) and 7 individuals suffering from isolated severe MBL deficiency. The sex and age matched control group had a mean age of 45.31 ( ± 10.4), that of the IEI/MBLdef group was 38.93 ( ± 13.87) years. In the IEI/MBLdef group, 11 patients were diagnosed with CVID, two patients with XLA, one patient with WHIM syndrome, one with an unreported deleterious mutation in the *ifngr1* gene (NM_000416.2:c.110T>C) as well as three patients with autoinflammatory disorders (two patients with CAPS, one with Muckle-Wells syndrome). Detailed information on the diagnosis and underlying autoimmune phenomena and infectious disease complications during the course of disease and immunomodulatory drugs as well as anti-infective drugs at the time of the blood collection are summarized in **Table 1**.

**Table 1:** Patient characteristics at baseline and autoimmune phenomena/infectious complications during the course of disease.

Patient no.	Age (y)	Gender	Underlying diagnosis	Diagnostic criteria	Autoimmune phenomena during the course of disease	Infectious complications during the course of disease	IgRT (g/month)	Trade name of IgRT	Immunomodulator/antibiotic
1	56	F	CVID	IgG: 527 IgA: 89.5 IgM: < 5.2	arthralgia, arthritis, fatigue, myalgia, splenomegaly, IBD-like	chronic gastritis	no		0
2	33	M	CVID	IgG: 353 IgA: 26.8 IgM: 34.9	IBD-like, history of splenectomy due to ITP	recurrent pneumonia, St.p. pancreatic abscess, aspergillosis	31	gammanorm^®^	0
3	30	F	CVID	CD19^+^: 65 IgG2 145 Anti pneumococc-al IgG 1:135 after vaccination	Sjögren´s syndrome, lymphadenopathy	recurrent pneumonia, recurrent diarrhea	no		0
4	21	M	CVID	IgG: 549 IgG1: 277.97 IgA: 110 IgD^+^CD27^+^: 10 IgD^-^CD27^+^: 10	none	recurrent pneumonia	no		0
5	41	F	CVID	IgG: < 195 IgA: < 6.3	SLE-like, fatigue, leucopenia, lymphadenopathy, hepatomegaly, splenomegaly, history of CNS vasculitis	recurrent cystitis	24	Hizentra^®^	Hydroxychloroquine
6	46	F	CVID	IgG: < 195 IgA: < 7.2	none	recurrent pneumonia	20	privigen^®^	0
7	29	F	CVID	IgG: 206, IgA: <7.0	arthralgia, arthritis, fatigue, GLILD, hepatomegaly, IBD-like, lymphadenopathy, leucopenia, splenomegaly, thrombocytopenia,	pneumonia, recurrent cystitis, pulmonary aspergillosis	48	Hizentra^®^	Rituximab
8	23	F	CVID	IgG: 167 IgA: <7.0	none	chronic sinusitis	33	gammanorm^®^	Minocyclin (St.p.)
9	58	F	CVID	IgG: 376 IgA: 130 IgM: 31.6	arthralgia, myalgia, pneumonitis, fatigue	bronchitis	no		0
10	53	M	CVID	IgG: 286 IgA: < 6.8	fatigue, IBD-like	pneumonia,	40	gammanorm^®^	0
11	60	M	CVID	IgG: < 195 IgA: < 33	arthralgia	bronchitis, sinusitis	20	privigen^®^	0
12	20	M	XLA	genetic	none	bronchitis, pneumonia, chronic hepatitis B	20	HyQvia^®^	Entecavir
13	38	M	XLA	genetic	none	chronic sinusitis, chronic otitis	16	privigen^®^	0
14	26	F	WHIM	genetic	leucopenia, splenomegaly	recurrent pneumonia, bronchitis, otitis, warts/HPV	16	Hizentra^®^	0
15	39	F	Mutation in IfngR1	genetic	rheumatoid arthritis, fatigue	recurrent pneumonia, bronchitis, otitis, chronic infection with M.avium (lung and CNS)	no		Salazopyrin, Dexamethason, Clarithromycin, Rifampicin, Tezdizolid, Moxifloxacin
16	40	M	Muckle-Wells syndrome (MWS)	genetic	arthralgia	none	no		Hydroxychloroquine
17	42	F	CAPS	genetic diagnosis by third party	arthralgia, fatigue, fever, myalgia, pleurisy, rash, lymphadenopathy, IBD-like, aseptic meningitis	recurrent oral ulcers, chronic gastritis, recurrent pyelonephritis	no		Canakinumab
18	19	M	CAPS	genetic	fatigue, myalgia, rash	none	no		Canakinumab
19	52	F	MBL-deficiency and selective deficiency in pneumococcal-al antibody response	pneumococcal 1:128, no dynamic after vaccination MBL 16.3	IBD-like, bronchiectasis	bronchitis, sinusitis, chronic gastritis	no		0
20	20	M	MBL-deficiency	MBL <0.5	none	none	no		0
21	39	F	MBL-deficiency	MBL <0.5	pericarditis	none	no		0
22	49	F	MBL-deficiency	MBL 29.8	none	recurrent pneumonia, sinusitis	no		0
23	25	F	MBL-deficiency	MBL <0.5	arthralgia, myalgia, lymphadenopathy	recurrent oral ulcers	no		0
24	41	F	MBL-deficiency	MBL <0.5	fatigue, rash, Raynaud´s phenomenon, lymphadenopathy, IBD-like	recurrent pneumonia, sinusitis, recurrent oral ulcers, chronic gastritis	no		0
25	39	F	MBL-deficiency	MBL <0.5	fatigue, myalgia, rash, IBD-like	chronic gastritis, recurrent herpes simplex infection, recurrent cystitis, recurring abscesses	no		0
26	69	F	MBL-deficiency	MBL <0.5	none	bronchitis, sinusitis, recurrent cystitis	no		0

The column “diagnostic criteria” shows the markers of immunodeficiency at the time of initial diagnosis. F, female; M, male; IgRT (g/month), Immunoglobulin replacement therapy in gram per month. IgG/IgM/IgA values are displayed in mg/dl. Cell counts are expressed in absolute numbers per microliter (cell/µl) and MBL levels in nanogram per microliter (ng/mL). The cutoffs/normal ranges are the following: IgG: 700-1600 mg/dL, IgG1: 280-800 mg/dL, IgG2: 169-786 mg/dL, IgA 70-400 mg/dL, IgM: 40-230 mg/dL, MBL: >300 ng/ml, CD19^+^: 100-500 c/µl; IgD^+^ CD27^+^ non–class-switched memory B cells: 10-110 c/µl; IgD^-^ CD27^+^ class-switched memory B cells: 10-80 c/µl, anti pneumococcal IgG 1:200.

Ten patients, suffering from CVID, XLA or WHIM, routinely received immunoglobulin replacement therapy (IgRT) of a average 26.8g per month ( ± 10.3g). Seven patients received the substitution therapy *via* the subcutaneous route, three patients intravenously (**Table 1**). Hence, there was no significant difference in the total amount of IgG levels between patients suffering from predominant hypogammaglobulinemia, autoinflammatory syndromes or MBL deficiency (**Table 2**). Consistent with the diagnoses, we detected significant differences in the absolute numbers of class-switched B lymphocytes in patients with hypogammaglobulinemia when compared to the other groups of diseases, while the absolute numbers of CD19^+^ B cells were not affected. In contrast we did not detect any differences in the number of CD4^+^ T cells and CD8^+^ T cells when we compared the different patient groups (**Table 2**).

**Table 2:** Comparative analysis of serological and cellular data of patients with hypogammaglobulinemia (with IgRT), autoinflammatory disorders and MBL-deficiency.

	Hypogammaglobulinemia (with IgRT)	Auto-inflammatory disorders	MBL-deficiency	p-value
N	14	3	7	
IgG (mg/dl) (NR:700-1400 mg/dl)	923 ( ± 404)	1367 ( ± 382)	1032 ( ± 155)	0.158
CD19^+^ B cells (c/µl) (NR: 100-500 c/µl)	167 ( ± 134)	183 ( ± 67)	216 ( ± 100)	0.687
IgD^+^ CD27^+^ non–class-switched memory B cells (c/µl) (NR:10-110 c/µl)	28 ( ± 42)	13 ( ± 6)	60 ( ± 44)	0.165
IgD^-^ CD27^+^ class-switched memory B cells (c/µl) (NR:10-80 c/µl)	9 ( ± 10)	17 ( ± 12)	64 ( ± 33)	**<0.001**
CD21^+^ B cells (c/µl) (NR:6-310 c/µl)	155 ( ± 127)	180 ( ± 61)	212 ( ± 94)	0.554
CD4^+^ T cells (c/µl) (300-1400 c/µl)	648 ( ± 302)	490 ( ± 272)	833 ( ± 342)	0.251
CD8^+^ T cells (c/µl) (NR:200-900 c/µl)	408 ( ± 290)	247 ( ± 159)	497 ( ± 211)	0.388

Patients number 15 and 19 were excluded because the diagnosis was not clearly attributable to a specific group. All values show the mean and standard deviation. Group differences were calculated using one-way ANOVA. Statistical significance defined by p ≤ 0.05 is highlighted. NR, Normal range. Statistical significance defined by p ≤ 0.05 is highlighted.

### Humoral immune responses to COVID-19 vaccination

**Figure 1:**
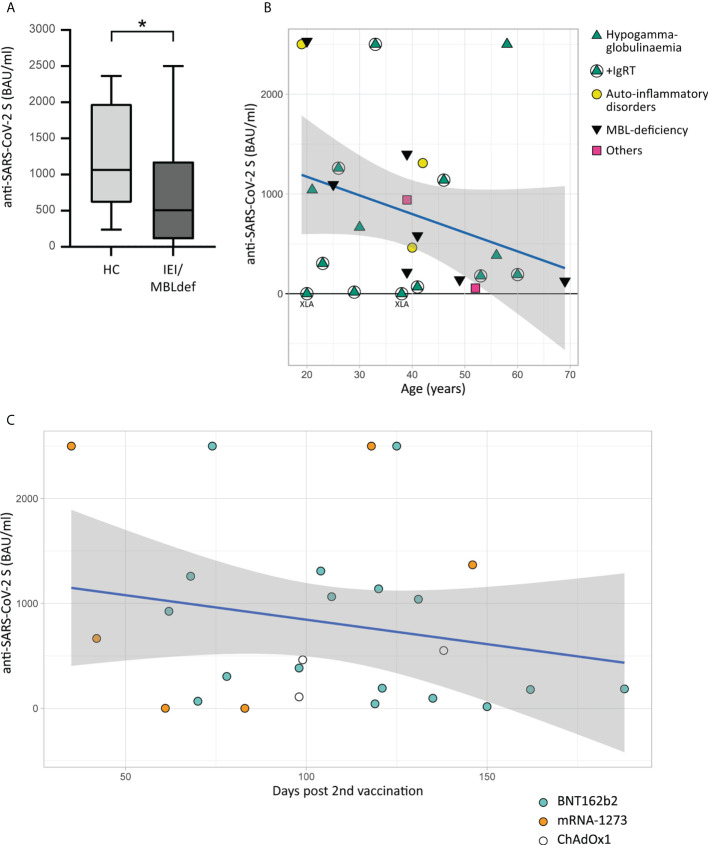
Humoral immune response to COVID-19 vaccination. Antibodies to the receptor-binding domain (RBD) of the viral spike (S) protein were determined using an anti-SARS-CoV-2 immunoassay. Values below 0.4 BAU/ml were defined as 0. **(A)**. Boxplot with IQR and maximum and minimum values whiskers. Comparison of anti-SARS-CoV-2 S levels of 26 matched healthy controls and the IEI/MBLdef group consisting of 26 patients. The asterisk (*) indicates a p<0.05. **(B)** Scatter plot of antibody levels to the RBD of the S protein (Y-axis) of 26 patients grouped into four classes and the age of the patients (x-axis) in years with a linear regression line including a 95% CI. Green triangles: patients with hypogammaglobulinemia. Patients receiving IgRT are marked with circles. Yellow circles: patients with auto-inflammatory disorders. Black inversed triangles: patients with MBL deficiency. The patient groups were defined by their predominant serological results as described in **Table 2**. Patients number 15 and 19 are highlighted with pink squares. The two patients with XLA showed values of anti-SARS-CoV-2 S antibodies below 0.8 BAU/ml. **(C)** Scatter plot shows the antibody levels to the RBD of the S protein in relation to time after vaccination of the 26 patients grouped into four classes and the type of vaccine with a linear regression line including a 95% CI.

Antibodies against the SARS-CoV-2 RBD of the S protein were analyzed after the second dose of COVID-19 vaccination. Individuals with IEI/MBLdef displayed a significantly lower anti-SARS-CoV-2 S antibody level when compared to the matched healthy control group (813.8 BAU/ml ( ± 836.3) vs. 1296.5 BAU/ml ( ± 682.9), p=0.0299, **Figure 1A**).

IEIs are a highly heterogenous group of diseases including multiple different monogenetic diseases. To gain further insights into this cohort, we divided the patients into three groups regarding their dominant phenotype as described in **Table 2**. Patients number 15 and 19 were excluded from the analysis since they did not classify for one specific group. It has been previously demonstrated, that the age of the patient is the most important factor for the level of vaccine response (24). In line with this we observed a trend towards lower anti-SARS-CoV-2 S antibody levels with higher patient age (Spearman, r= -0.2285, p= 0.2616. **Figure 1B**). We did not detect any apparent effect of the underlying disease on the production of anti-SARS-CoV-2 S antibodies, except for the two XLA patients (**Figure 1B**). Furthermore it has been demonstrated that antibody levels decline over time (25, 26). This finding was confirmed in the IEI/MBLdef cohort with the detection of a negative correlation between the levels of anti-SARS-CoV-2 S antibodies and the time passed since vaccination. However, this finding did not reach statistical significance (Spearman, r=-0.1021, p-value = 0.6198, **Figure 1C**). In total, 17 patients received two vaccinations with BNT162b2 (Corminaty^®^, Pfizer BioNTech), six patients were vaccinated twice using mRNA-1273 (Spikevax^®^, Moderna) and three patients received two doses of the vector vaccine ChAdOx1 (Vaxzevria^®^, Astra-Zeneca) (**Figure 1C**). No single vaccine was superior in terms of their effect on the levels of anti-SARS-CoV-2 S antibody production (Kruskal-Wallis chi-squared = 0.4268, df = 2, p-value = 0.8078). High levels of anti-SARS-CoV-2 S antibodies are considered to be associated with protection against SARS-CoV-2 infection (26, 27). However, as a result of frequent mutations of the SARS-CoV-2 virus, no single protective cutoff can be given. We thus decided to identify an anti-SARS-CoV-2 S antibody level which would help to separate healthy vaccine responders from those with severely impaired vaccine response. Based on previously published vaccine response in healthy controls and taking into account the relatively late date of sampling in our cohort we defined a level of 100 BAU/ml of anti-SARS-CoV-2 S antibodies as discriminator (26, 27). While our healthy control group showed a diverse but nevertheless 100% response rate with anti-SARS-CoV-2 S levels ranging from 238 BAU/ml up to 2363 BAU/ml (median 1064 BAU/ml), (IQR 680-1918), we observed a rather limited serological response to the vaccination in our IEI/MBLdef cohort. Overall, a total of six patients fell below the 100 BAU/ml threshold, including two patients suffering from XLA. Very low levels of anti-SARS-CoV-2 S antibodies were detected in two CVID patients, of whom one received Rituximab four months after the vaccination. Interestingly, low anti-SARS-CoV-2 S titers were also detected in two MBL-deficient patients (patient number 19 and 26). In both patients, we did not detect any irregular numbers of B or T cell subsets (data not shown). In summary, when applying the cut-off of 100 BAU/ml, we observed immunogenicity of the second vaccination in 20 out of 26 patients, while all 26 healthy subjects achieved antibody levels well above this threshold.

### Neutralizing activity against SARS-CoV-2

**Figure 2:**
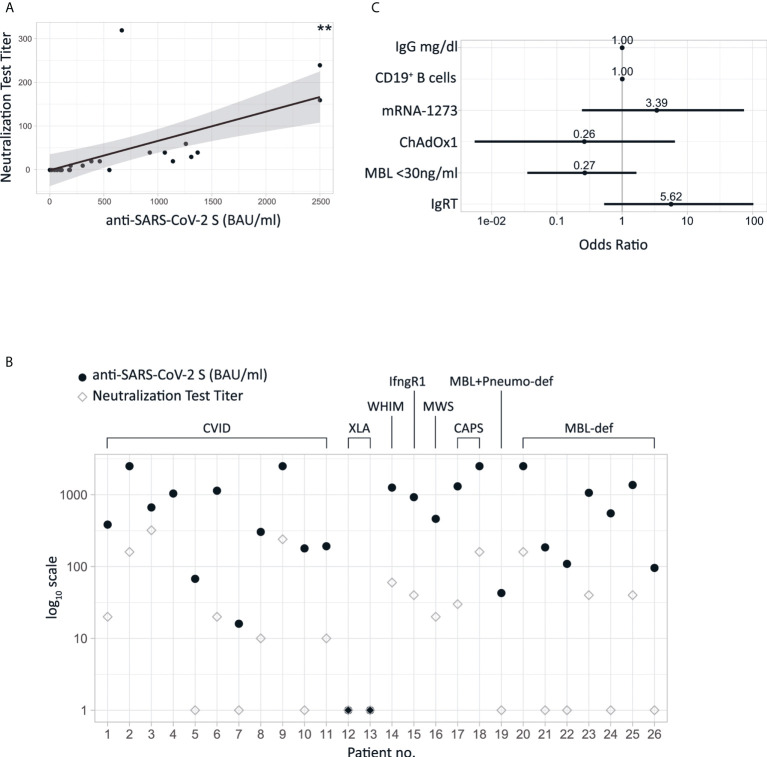
**(A)** Scatter plot of neutralizing test titers and anti-SARS-CoV-2 S with linear regression line. The double asterisk indicate a p<0.001. **(B)** Levels of anti-SARS-CoV-2 S in combination with titers of neutralizing antibodies are shown for the individual patients **(C)** ORs of logistic regression analyses assessing neutralizing antibody titers against SARS-CoV-2Virus. MBL-deficiency was defined as MBL <30ng/ml. IgRT: Immunoglobulin replacement therapy. Reference vaccine to the ORs of ChAdOx1 and mRNA-1273 is BNT162b2.

Neutralizing capability of antibodies against SARS-CoV-2 after the second vaccination was assessed in 25 patients. We detected a significant positive correlation between levels of anti-SARS-CoV-2 S antibodies and the respective neutralization test titers (Spearman, r=0.8823, p<0,001, **Figure 2A**). The level of anti-SARS-CoV-2 S antibodies was not predictive for neutralizing capacity in all patients, since some individuals showed relatively high neutralizing antibody titers in relation to their serum anti-SARS-CoV-2 S antibody levels, while others – in spite of high anti-SARS-CoV-2 S antibody levels – did not. We observed this phenomenon independent of the underlying disease (**Figure 2B**). A multivariable logistic regression analysis demonstrated that the ChAdOx1 vaccine showed a trend in favor of non-neutralizing capability (OR 0.26, 95% CI 0.0055 - 6.4343), whereas the mRNA-1273 (OR 3.39, 95% CI 0.2421-73.6718) vaccine showed a trend towards higher likelihood of mounting neutralizing activity, with the BNT162b2 vaccine as reference (**Figure 2C**). Furthermore, there was no significant association between the total amount of IgG (OR 1, 95% CI 0.9962 - 1.0031), the number of CD19^+^ B cells (OR 1, 95% CI 0.9939 - 1.017) or the MBL levels (OR 0.27, 95% CI 0.0352 - 1.6530) with titers of neutralizing antibody as reference (**Figure 2C**).

### Influence of IgRT

Interestingly four patients with immunoglobulin replacement treatment displayed detectable SARS-CoV-2 nucleocapsid antibodies without ever reporting an active SARS-CoV2 disease or related symptoms nor being positive PCR-tested for SARS-CoV-2 in regularly performed checkups (data not shown). This gives rise to the speculation that antibodies might be artificially transferred to substituted patients. Multivariable logistic regression analysis revealed that the presence of neutralizing antibodies after vaccination was associated with immunoglobulin substitution therapy, however a statistical significance was not reached (OR 5.62, 95% CI 0.5304-102.7768) (**Figure 2C**). To further evaluate the impact of IgRT we analyzed eight batches of three different preparations of IgRT which were administered to the patients for a maximum of six weeks prior to the blood draw (privigen^®^ P100320533 and P100313729, Hizentra^®^, P100318336, P10032804, P100311233 and gammanorm^®^ M032C8605 and M105A8601). Interestingly, anti-SARS-CoV-2 S antibodies were present in all batches of immunoglobulins although at varying levels (data not shown). Of note, in the sera of the two XLA patients, both receiving IgRT, we did not show any anti-SARS-CoV-2 S antibodies.

### T cell-mediated immune response to COVID-19 vaccination

**Figure 3:**
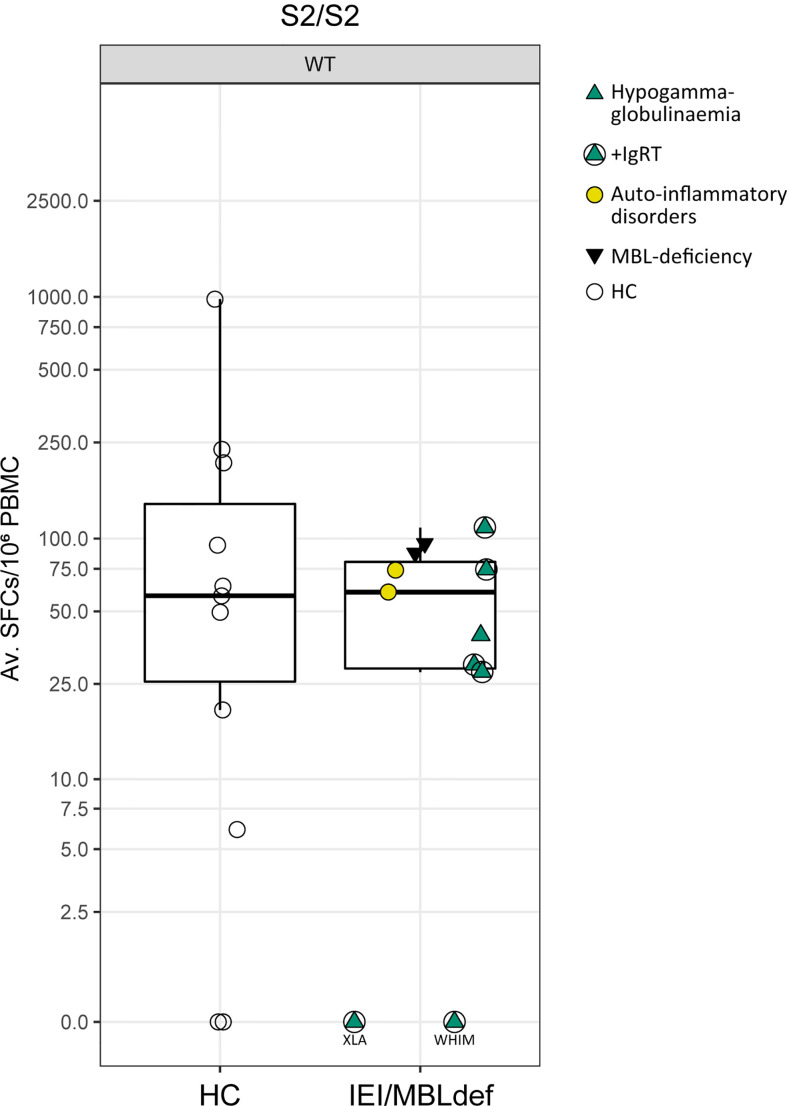
SARS-CoV-2 specific T-cell responses. SARS-CoV-2 specific T cell responses were detected by ELISpot assay from peripheral blood mononuclear cells (PBMCs) stimulated with wild type (WT) spike unit S1 and S2 peptide pools after the second vaccination. Composite ELISpot results from 11 patients and 11 HCs; Av SFCs/10^6^ PBMC: Average of spot-forming cells per 10^6^ peripheral blood mononuclear cells. Circles: healthy controls, green triangles: patients with hypogammaglobulinemia. Patients receiving IgRT are marked with circles. Yellow circles: patients with auto-inflammatory disorders. Black inversed triangles: patients with MBL deficiency.

Especially in light of the rapid mutation frequency of the SARS-CoV-2 virus, T cell response seems to be a key in generating a robust immune response (28, 29). To investigate whether the IEI/MBLdef patients mounted a SARS-CoV-2 T cell specific response, we analyzed PBMCs from eleven patients in comparison to PBMCs of healthy controls. With the exception of the patients suffering from WHIM and XLA, nine patients (82%) had a detectable cellular response to the S peptide pools (S1/S2) after two vaccinations. In the HC cohort we detected a similar response rate of 82% (9 out of 11). The median response in the IEI/MBLdef cohort was similar (60 SFCs/10^6^ PBMCs, IQR 29 – 80.25), when compared to healthy controls (58 SFCs/10^6^ PBMCs, IQR 26.5 – 150, p = 0.267 **Figure 3**). We did not detect a significant correlation between T cell response and anti-SARS-CoV-2 S IgG (r = 0.0157, Spearman).

### Reactogenicity

**Figure 4:**
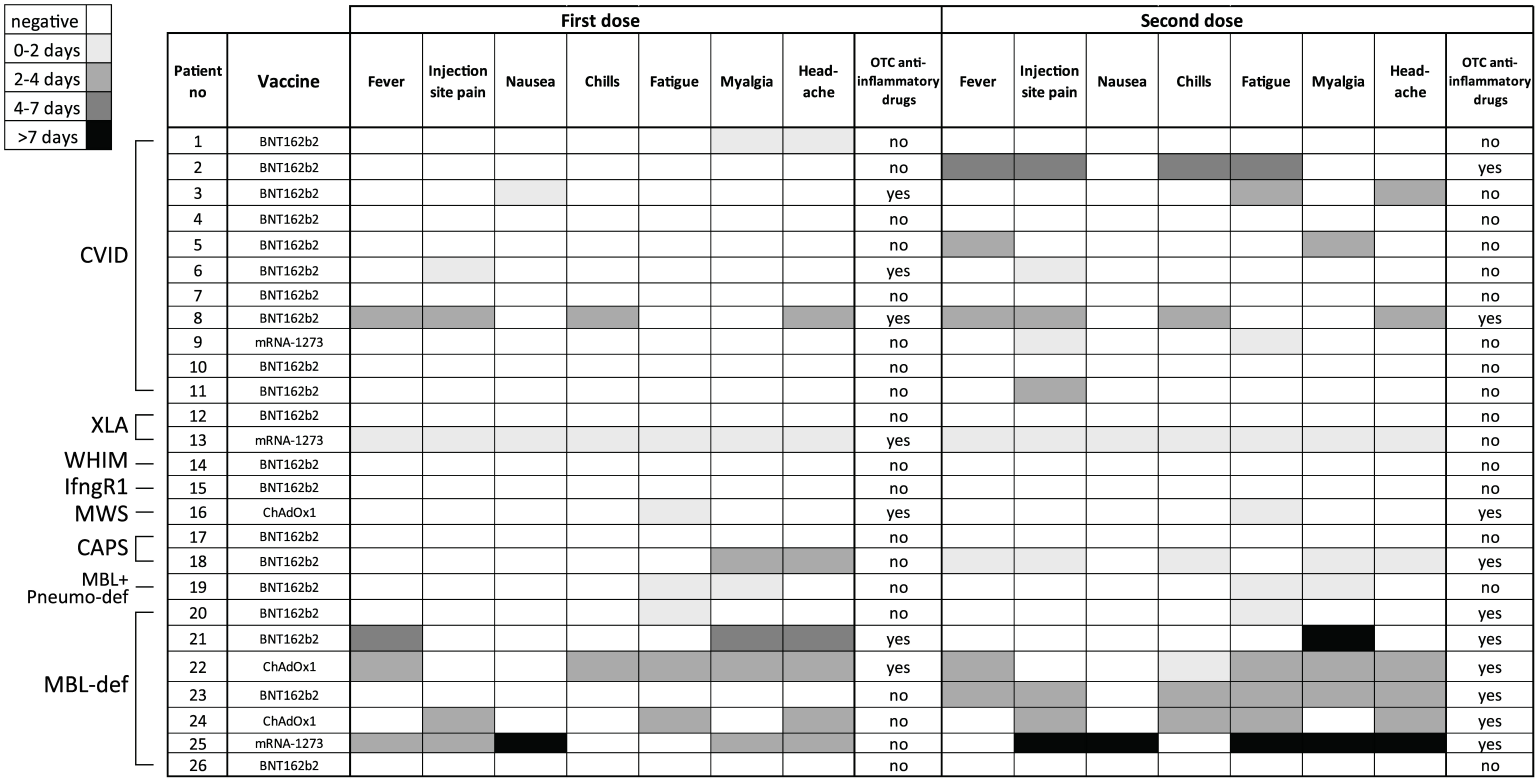
Summary of the duration of the reactogenicity and use of over-the-counter anti-inflammatory drugs in individual patients vaccinated with the indicated vaccines. Duration of the symptoms were structured and gray-scale coded into four classes.

**Figure 5:**
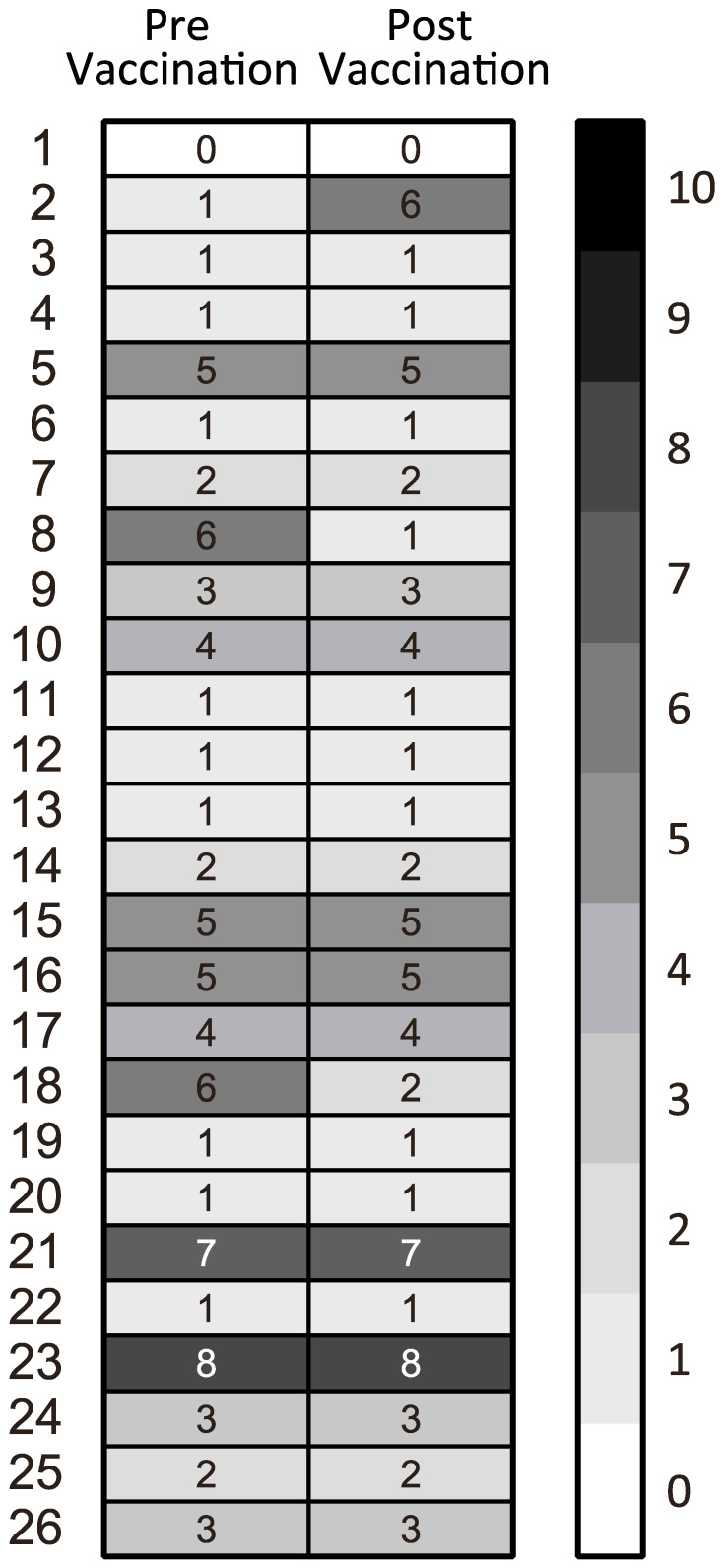
Heatmap of the dynamics of disease activity evaluated by using a patient global assessment (NRS) before the first vaccination and after the second vaccination.

In general, patients did not report any prolonged or serious adverse events in relation to vaccination. Only two patients with MBL deficiency reported adverse reactions lasting longer than seven days. Between the first and the second vaccination, increased reactogenicity especially fever, chills, pain at site of injection and fatigue, was reported regardless of the vaccine used. Accordingly, an increased use of over-the-counter (OTC) anti-inflammatory drugs between the first and second vaccination (from 7 to 10 patients) was noted. Neither the WHIM patient nor the patient suffering from IFN-gamma receptor deficiency reported any adverse reactions while at the same time developing a robust immune response (**Figure 4**). When the patients were interviewed using a PGA regarding their subjective disease activity after cessation of the acute vaccine response, only one patient reported an increase of the disease activity, while, remarkably, two patients reported an improvement of their underlying disease. However, in one of these patients (with diagnosed CAPS) the immunomodulatory therapy with canakinumab was initiated after the first vaccination (**Figure 5**). All other patients did not describe an impact of the vaccination on the activity of their illness or triggering of selective autoimmune phenomena (data not shown). This data might suggest very little to no effects of vaccination on the course of underlying disease in patients suffering from IEIs or MBL deficiency.

## Discussion

In this study we provide evidence that patients with IEI or MBL deficiency show the capacity to develop a detectable immune response to COVID-19 vaccines. The immunization was well tolerated with only minor adverse events which is in line with previous data on COVID-19 vaccination in healthy individuals. Importantly, we did not observe any major or rare adverse events that have been described for COVID-19 vaccines. Thus, regarding safety, our data is in line with two smaller studies that included patients with IEIs (8, 30).

In the IEI cohort, side effects after both doses of vaccination were rare and mild. In contrast, we observed an accumulation of side effects and prolonged duration of symptoms in individuals with MBL deficiency. In comparison to the other patients, this group used considerably more OTC anti-inflammatory drugs, especially after the second vaccination. Further research into this phenomenon may be warranted. Previously undescribed, we show, that the activity of an underlying autoimmune pathology in general did not increase in response to the vaccination independently of the diagnosis. In respect of reports discussing an autoimmune trigger by SARS- CoV-2 infection and by SARS-CoV-2 vaccination our study thus contributes important observations to the data recently presented by Dotan et al. (31) The amount of the humoral response to COVID-19 vaccination was mainly driven by factors that are also found in the general population such as age and, to a lesser extent, vaccine type (32). The majority of patients was able to produce anti-S-antibodies, suggesting that they possess the necessary B cell repertoire to do so. Patients vaccinated with the ChAdOx1 vaccine showed a trend towards lower specific antibody concentrations. In general, there was a positive correlation between anti-S-antibody levels and neutralizing antibody titers. Nevertheless, this was not true for every individual patient. This certainly highlights the importance of an individual assessment of the neutralizing capability of COVID-19 vaccines in patients suffering from IEI/MBLdef by virus neutralization tests or with the help of molecular interaction tests, respectively (33). Additionally, the low neutralizing titers in several patients clearly stress the need for monitoring of antibody levels as well as early revaccination in this population.

In line with previous reports of increasing amounts of anti-SARS-CoV-2 S antibodies over the time of the pandemic (34), we also were able to demonstrate the presence of anti-SARS-CoV-2 S antibodies in all tested lots of IgRT preparations. Hence, Ig replacement therapy might represent an, albeit limited, prophylaxis strategy for patients with primary immunodeficiencies that affect antibody production. In contrast to this finding however, in line with the defect and previous reports (35) the sera of the two XLA patients did not show increased anti-SARS-CoV-2 S antibodies, implicating that IgRT may distort the results of anti-SARS-CoV-2 S IgG measurement only to a very limited extend.

Interestingly, in 4 out of 10 patients with IgRT anti-nucleocapsid antibodies were detectable without proven infection *via* clinical reports or PCR testing. This could either be a representation of sufficient protection against symptomatic COVID-19 disease through IgRT, or could be an indicator of a possible transferal of anti-nucleocapsid antibodies during IgRT. Which in term would make a “status post infection” indistinguishable from “contamination by IgRT”. Nucleocapsid tests therefore might not be reliable for the detection of former infection in IgRT recipients.

MBL deficiency is not classified as IEI according to the most recent IUIS classification (1). In line with this, anti-SARS-CoV-2 S antibody response in the MBLdef group was adequate, with however low neutralization titers in subjects 19, 21, 22, 24 and 26. Surprisingly our study showed increased adverse reactions in these patients compared to the IEI cohort. This may either be due to the relatively unimpaired immune response in these patients, or may be connected to the increased incidence of MBLdef in patients suffering from chronic fatigue syndrome/myalgic encephalomyelitis (ME/CFS) which is characterized by headaches, myalgia and arthralgia (36). Further research into a possibly increased reactogenicity in the MBL cohort is essential. Most patients in our cohort had a T cell response comparable to healthy controls implicating that though humoral immune response may be compromised in some patients, invasive COVID infection may be limited through cellular response in these patients (37, 38). Of note, the patient suffering from WHIM syndrome, although generating a robust humoral response, did show a T cell response similar to that of the XLA patient. While the low T cell response in itself is expected in WHIM patients (39), the high antibody level would imply that the WHIM patient had received SARS-CoV-2 specific antibodies with her IgRT, while the XLA patients did not. This would be supported by the varying amount of anti-SARS-CoV-2 anti S antibodies found in the IgRT samples. In contrast to this, four other patients undergoing regular IgRT did develop a sufficient T cell response. Additionally, in regards to this finding it should be considered, that two of the healthy controls did also not show any T cell response to the vaccination. In line with previous reports, most of our patients developed a robust T cell response, although we were – probably as a consequence of the small sample size – not able to detect a worse response in CVID patients (40). We were not able to show a correlation between T cell response and anti-SARS-CoV-2 S IgG levels. When excluding the WHIM patient, the correlation improved only marginally to a correlation coefficient of 0.233.

We are aware that our study is limited by the small sample size of patients with diverse underlying rare diagnoses. More extensive trials will certainly be needed to assess and compare immunogenicity and reactogenicity to different COVID-19 vaccination regimes. Although we do not have the power to make a clear correlation with vaccine type and efficacy, our study provides data on the efficacy of three different COVID-19 vaccines. Moreover, the long interval of two to five months between the second vaccination and serum sampling suggests that COVID-19 vaccination is able to generate sustained antibody responses in this patient cohort. Unfortunately T cell response data could not be generated for all participants. Noteworthy, neither CVID patients, nor individuals suffering from autoinflammatory diseases showed a triggering of an (underlying) inflammatory condition through vaccination.

In summary we describe vaccination responses in a heterogenous group of IEI patients. Vaccination of these patients against SARS-CoV-2 appears to be safe and most patients are able to develop sustained vaccine-specific antibody as well as cell based responses. Individual assessment and monitoring of vaccine response, however, seems prudent.

## Data availability statement

The raw data supporting the conclusions of this article will be made available by the authors, without undue reservation.

## Ethics statement

The studies involving human participants were reviewed and approved by Ethikkommission der Medizinischen Universität Wien. The patients/participants provided their written informed consent to participate in this study.

## Author contributions

LG: conceptualization, data curation, formal analysis, investigation, validation, methodology, writing – original draft; DM: data curation, formal analysis, software, visualization, writing – review and editing; KG-P: investigation, validation, methodology, writing – original draft; KS: methodology, resources, writing – review and editing; HH: methodology, resources; LS: data curation, formal analysis, software; TD: formal analysis, software, visualization; FK: visualization; ST: data curation, conceptualization, project administration; DA: resources, supervision, project administration; HB: resources, supervision, project administration; MB: conceptualization, resources, supervision, funding acquisition, project administration, writing – review and editing; WP: methodology, resources, writing – review and editing; EF-W: investigation, validation, methodology, writing – original draft; CS: investigation, validation, methodology, writing – original draft; MV: conceptualization, data curation, validation, formal analysis, supervision, investigation, methodology, writing – original draft. All authors contributed to the article and approved the submitted version.

## Funding

This work was performed as part of the research obligation to the Medical University of Vienna. The University had no involvement in study design or data collection, analysis or interpretation, neither did it influence the writing or the submission process.

## Acknowledgments

The expert technical help of Ulrike Körmöczi, Teresa Oberhofer, Arno Rottal, Lisa Leonhartsberger, Karim El-Gedawi, Melanie Feichtner and Jutta Hutecek is acknowledged.

## Conflict of interest

DA is a member of the editorial board of “Annals of the Rheumatic diseases”.

The remaining authors declare that the research was conducted in the absence of any commercial or financial relationships that could be construed as a potential conflict of interest.

## Publisher’s note

All claims expressed in this article are solely those of the authors and do not necessarily represent those of their affiliated organizations, or those of the publisher, the editors and the reviewers. Any product that may be evaluated in this article, or claim that may be made by its manufacturer, is not guaranteed or endorsed by the publisher.
